# Treatment outcomes of stereotactic body radiation therapy for primary and metastatic sarcoma of the spine

**DOI:** 10.1186/s13014-023-02346-w

**Published:** 2023-09-22

**Authors:** Eunji Kim, Mi-Sook Kim, Eun Kyung Paik, Ung-Kyu Chang, Chang-Bae Kong

**Affiliations:** 1 Department of Radiation Oncology, Seoul Metropolitan Government - Seoul National University Boramae Medical Center, Seoul, Republic of Korea; 2 Department of Radiation Oncology, Korea Institute of Radiological and Medical Sciences, Seoul, Republic of Korea; 3 Department of Neurosurgery, Korea Institute of Radiological and Medical Sciences, Seoul, Republic of Korea; 4 Department of Orthopedic Surgery, Korea Institute of Radiological and Medical Sciences, 75, Nowon-ro, Nowon-gu, Seoul, 01812 Republic of Korea

**Keywords:** Sarcoma, Spine, Stereotactic body radiation therapy, Oligometastasis

## Abstract

**Purpose:**

This study evaluated the treatment outcomes of spine stereotactic body radiation therapy (SBRT) in sarcoma patients.

**Materials and methods:**

A total of 44 sarcoma patients and 75 spinal lesions (6 primary tumors, 69 metastatic tumors) treated with SBRT were retrospectively reviewed between 2006 and 2017. The median radiation dose was 33 Gy (range, 18–45 Gy) in 3 fractions (range, 1–5) prescribed to the 75% isodose line.

**Results:**

The median follow-up duration was 18.2 months. The 1-year local control was 76.4%, and patients treated with single vertebral body were identified as a favorable prognostic factor on multivariate analyses. Progression-free survival at 1 year was 31.9%, with the interval between initial diagnosis and SBRT and extent of disease at the time of treatment being significant prognostic factors. The 1-year overall survival was 80.5%, and PTV and visceral metastases were independently associated with inferior overall survival.

**Conclusion:**

SBRT for spinal sarcoma is effective in achieving local control, particularly when treating a single vertebral level with a limited extent of disease involvement, resulting in an excellent control rate. The extent of disease at the time of SBRT is significantly correlated with survival outcomes and should be considered when treating spine sarcoma.

**Supplementary Information:**

The online version contains supplementary material available at 10.1186/s13014-023-02346-w.

## Introduction

Sarcomas are rare cancers that originate from mesenchymal cells and represent a heterogeneous group with various histologies [1, 2]. Although radical surgery and radiotherapy are initially performed in patients with sarcoma, a significant number of patients eventually develop distant metastases [3, 4]. Spinal metastases, among the distant metastases, can lead to severe pain and disability, significantly affecting the management of the disease. A multidisciplinary approach involving surgery, radiotherapy, and chemotherapy is employed to treat patients with metastatic spinal diseases [5, 6].

For achieving effective local control (LC) of spinal metastases, the options of surgical resection and radiotherapy should be considered. While complete resection of the metastatic tumor has shown a high rate of tumor control, it may be limited due to potential complications. Radiotherapy, on the other hand, offers different modalities including conventional radiotherapy and stereotactic body radiation therapy (SBRT). Among these, SBRT is often preferred for the treatment of spinal metastases, as it allows for the delivery of high radiation doses, resulting the effective LC while minimizing associated toxicity [3].

Previous studies have reported the clinical effectiveness of stereotactic radiotherapy for metastatic lesions, including spine metastases [7–9]. These studies have primarily focused on common carcinomas such as breast, lung, colorectal, and prostate cancer. Although we are curious about the outcomes of spine SBRT in sarcoma, which is known to be radioresistant, only a few studies have been conducted due to its rarity [10–14]. At our institution, which serves as a single, prominent referral center for sarcoma, we previously reported the LC rate of spinal SBRT in sarcoma [15]. In particular, we anticipated that spinal SBRT could provide more substantial benefits compared to conventional radiotherapy for patients with primary, oligometastatic, or oligoprogressive disease. Since then, we have administered spine SBRT to a considerable number of patients. This study aims to further establish the efficacy of SBRT for spinal sarcoma and discern prognostic factors that may influence clinical outcomes through an analysis of recent clinical data.

## Materials and methods

### Patient

We conducted a retrospective review of medical records for patients who underwent spine SBRT for sarcoma between January 2006 and December 2017. The inclusion criteria were as follows: (1) histologically confirmed primary sarcoma, (2) primary, oligometastatic, or oligoprogressive disease, defined as a limited number of lesions (≤ 5), (3) the absence of neurologic deficits or spinal instability, and (4) no prior SBRT at the treatment site. All patients underwent SBRT for either definitive or salvage aim. Patients who did not have post-treatment hospital visit were excluded, and assessed through follow-up radiological evaluations. The time from initial diagnosis was calculated from the date of primary diagnosis to the start date of SBRT. This study was approved by the institutional review board of Korea Cancer Center Hospital.

### Radiation therapy

For SBRT, computed tomography (CT) simulation was performed with a 1.25 mm slice thickness. The patient was positioned in the supine position using a custom-made immobilization device, such as thermoplastic head mask or vacuum cushion. The gross tumor volume (GTV) was delineated on axial CT slices based on T1- and T2-weighted magnetic resonance imaging (MRI), and the planning target volume (PTV) margin was usually a 1–3 mm from GTV using the CyberKnife treatment planning system (Accuray Inc., Sunnyvale, CA, USA). The prescribed dose and fractionation were determined by the physicians. Treatment was delivered using CyberKnife (Accuray Inc., Sunnyvale, CA, USA), with the dose prescribed to the 75% isodose line. Treatment planning images are shown in Additional file 1: Fig. S1. Based on the linear-quadratic model and previous findings, a biological equivalent dose (BED) was calculated for the prescription based on the α/β ratio of 5 Gy for tumor effect (BED_5_) [16].

### Statistical analysis

LC was defined as the time to local failure at the treatment site. Adjacent recurrence was described at the level of the spine one above and below outside PTV. An event for progression-free survival (PFS) and overall survival (OS) was defined as any recurrence of lesions and death of a patient from any cause, respectively. Progression-free survival (PFS) and overall survival (OS) were measured from the initial date of SBRT to the occurrence of relevant events. Survival rates were estimated using the Kaplan–Meier method. Univariate analysis was performed using the log-rank test and Cox model. Variables with p-value less than 0.1 in univariate analysis were selected for multivariate Cox proportional hazard model with backward elimination method. A p-value of less than 0.05 was considered statistically significant. All statistical analyses were performed using R 4.2.1 (The R Foundation for Statistical Computing, Vienna, Austria).

## Results

### Patient and tumor characteristics

A total of 44 patients with 75 lesions were included in the study. The patient characteristics are summarized in Table 1. Of the patients, 30 were male and 14 were female, with a median age of 40 years (range, 14–76 years) at the time of their first SBRT. The median time from the initial diagnosis to SBRT was 20 months (range, 0–141 months). Osteosarcoma was the most common histology (n = 24, 54.6%). There were 16 patients with soft tissue sarcoma, including liposarcoma (n = 3), malignant peripheral nerve sheath tumor (n = 3), leiomyosarcoma (n = 3), pleomorphic sarcoma (n = 2), and fibrosarcoma (n = 1).

**Table 1:** Patient characteristics

Variable	Numbers
Number of patients	44
Median age, year (range)	40 (14–76)
Gender	
Men	30 (68.2%)
Women	14 (31.8%)
Histology	
Osteosarcoma	24 (54.6%)
Chondrosarcoma	3 (6.8%)
Ewing’s sarcoma	1 (2.3%)
Soft tissue sarcoma	16 (36.4%)
Liposarcoma, malignant peripheral nerve sheath tumor, and leiomyosarcoma	3 (6.8%, each)
Pleomorphic sarcoma	2 (4.5%)
Fibrosarcoma	1 (2.3%)

The tumor and treatment characteristics are presented in Table 2. Among the 75 lesions, 69 (92.0%) were metastatic diseases. The most common sites of metastasis were the thoracic spine (45.3%) and lumbar spine (24.0%). Two (2.7%) had been previously irradiated with conventional radiotherapy, and 16 (21.3%) had undergone surgery for mechanical stability or spinal cord decompression. Sixty lesions (80.0%) involved a single level, while 15 lesions (20.0%) involved 2 or 3 vertebral levels. At the time of SBRT, 49 lesions (74.5%) were presented with visceral metastases, 15 (20.0%) with solitary spine involvement, and 11 (14.7%) with multiple spine or bone metastases. The median PTV was 16.0 cc (range, 1.3–163.6 cc). The median prescription dose was 33 Gy (range, 18–45 Gy) delivered in three fractions (range, 1–5 fx), with a median BED was 100 Gy_5_ (range, 60–180 Gy_5_). The median PTV coverage was 98.8% (range, 93.6–100.0%).

**Table 2:** Treatment characteristics

Variable	Numbers
Number of treated lesions	75
Histology of lesions	
Osteosarcoma	43 (57.3%)
Chondrosarcoma	3 (4.0%)
Ewing’s sarcoma	2 (2.7%)
Soft tissue sarcoma	27 (36.0%)
Spinal disease status	
Primary	6 (8.0%)
Metastasis	69 (92.0%)
Prior local therapy	
Any surgery	16 (21.3%)
External beam radiotherapy	2 (2.7%)
Site of lesions	
Cervical	11 (14.7%)
Thoracic	34 (45.3%)
Lumbar	18 (24.0%)
Sacral	12 (16.0%)
Treated vertebral level	
Single	60 (80.0%)
2–3	15 (20.0%)
Extent of disease at the time of treatment	
Solitary spine involvement	15 (20.0%)
Multiple bone metastases	11 (14.7%)
Visceral metastases	49 (74.5%)
Median dose, Gy (range)	33 (18–45)
Median BED, Gy_5_ (range)	100 (60–180)
Fractionation	
Single	12 (16.0%)
2–5 fx	63 (84.0%)
Median PTV, cc (range)	16.0 (1.3–163.6)

*BED* biological equivalent dose, *PTV* planning target volume

### Local control

**Fig. 1:**
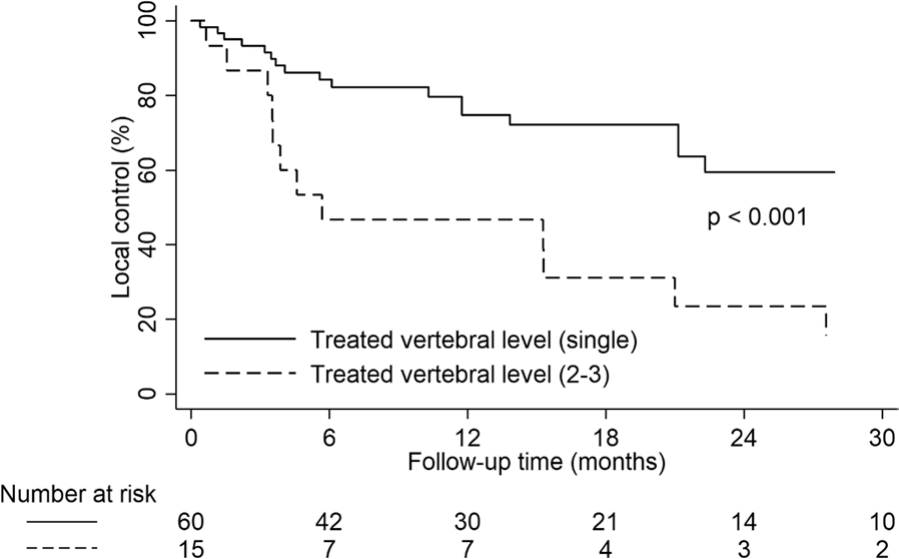
Local control rate according to the number of treated vertebral level

With a median follow-up time of 18.2 months (range, 2.4–153.7 months), locoregional recurrence occurred in 30 patients (30.7%), with 20 cases of local recurrence only, 3 cases of recurrence in both local and adjacent sites, and 7 cases of adjacent recurrence only. The 1-, 2-, and 3-year LC rates were 76.4%, 62.9%, and 54.4%, respectively. Univariate and multivariate analyses (Table 3) identified multiple vertebral levels (hazard ratio [HR] 3.031, 95% confidence interval [CI] 1.098–8365, p = 0.032) as an independent prognostic factor for LC (Fig. 1). Radiation dose was a statistically significant factor on univariate analyses but not significant on multivariate analyses.

**Table 3:** Univariate and multivariate analyses of local control

Variables	Univariate		Multivariate		
	1-year rate (%)	p-value	HR	95% CI	p-value
Gender					
Men	89.8	0.062			
Women	63.5				
Age (year)					
≤ 40	81.0	0.955			
> 40	79.7				
Histology					
Osteosarcoma	77.4	0.393			
Others	75.8				
Spinal disease status					
Primary	100.0	0.079			
Metastasis	77.8				
Any prior radiotherapy					
Yes	100.0	0.854			
No	75.7				
Any prior surgery					
Yes	79.8	0.297			
No	75.6				
Time from initial diagnosis (month, continuous)	–	0.811			
Site of lesions					
Cervical	63.6	0.184			
Thoracic	85.3				
Lumbar	71.8				
Sacral	71.4				
Treated vertebral level					
Single	80.9	< 0.001	1		0.032
2–3	60.0		3.031	1.098–8.365	
BED (Gy_5_, continuous)	–	0.031			
PTV (cc, continuous)	–	0.747			
Extent of disease at the time of treatment					
Solitary spine involvement	73.3	0.560			
Multiple bone metastases	72.7				
Visceral metastases	77.2				

*HR* hazard ratio, *CI* confidence interval, *BED* biological equivalent dose, *PTV* planning target volume

### Survival and prognostic factors

**Fig. 2:**
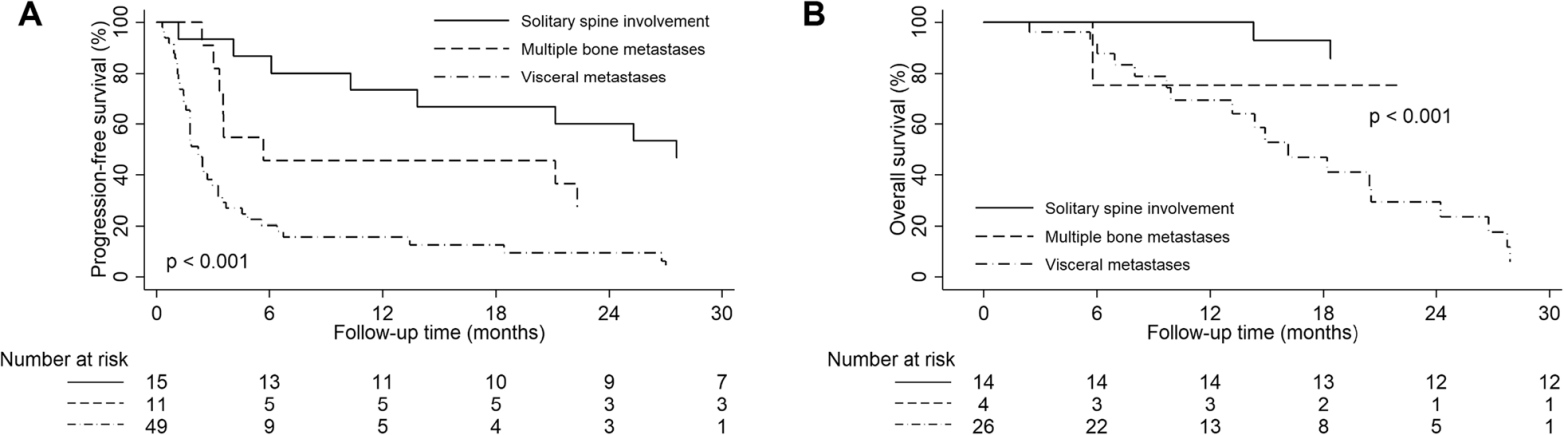
(**A**) Progression-free and (**B**) overall survival according to the extent of disease at the treatment time

The 1-, 2-, and 3-year PFS rates were 31.9%, 22.8%, and 15.2%, respectively. Univariate and multivariate analyses identified independent prognostic factors of survival outcomes (Table 4). Although univariate analyses showed that several factors were significant prognostic factors for PFS, on multivariate analysis, only time from initial diagnosis (HR 0.979, 95% CI 0.968–0.990, p < 0.001) and extent of disease at the time of treatment (solitary vs. multiple bone metastases, HR 6.853, 95% CI 2.205–21.296, p < 0.001; solitary vs. visceral metastases, HR 5.618, 95% CI 2.301–13.715, p < 0.001) remained significantly correlated with PFS. The 1-year PFS was 15.7% in patients with visceral metastases (Fig. 2A).

**Table 4:** Univariate and multivariate analyses of progression-free and overall survival

Variables	Progression-free survival					Overall survival				
	Univariate		Multivariate			Univariate		Multivariate		
	1-year rate (%)	p-value (uni)	HR	95% CI	p-value (multi)	1-year rate (%)	p-value (uni)	HR	95% CI	p-value (multi)
Gender										
Men	40.0	0.159				74.7	0.271			
Women	49.0					92.3				
Age (year)										
≤ 40	47.6	0.691				81.0	0.607			
> 40	38.0					80.5				
Histology										
Osteosarcoma	37.5	0.239				69.7	0.246			
Others	29.3					94.1				
Spinal disease status										
Primary	83.3	0.061				100.0	0.079			
Metastasis	27.2					77.8				
Time from initial diagnosis (month, continuous)	–	0.026	0.979	0.968–0.990	< 0.001	–	0.448			
Treated vertebral level										
Single	33.2	0.504				82.0	0.469			
2–3	26.7					75.0				
BED (Gy_5_, continuous)	–	0.897				–	0.194			
PTV (cc, continuous)	–	0.436				–	0.056	1.013	1.003–1.024	0.013
Extent of disease at the time of treatment										
Solitary spine involvement	73.3	< 0.001	1		< 0.001	100.0	< 0.001	1		< 0.001
Multiple bone metastases	45.5		6.853	2.205–21.296		75.0		5.991	0.993–36.133	
Visceral metastases	15.7		5.618	2.301–13.715		69.2		13.404	3.706–48.479	

*HR* hazard ratio, *CI* confidence interval, *BED* biological equivalent dose, *PTV* planning target volume

Regarding OS, the 1-, 2-, and 3-year OS rates were 80.5%, 54.9%, and 42.7%, respectively. Multivariate analyses determined that larger PTV volume (HR 1.013, 95% CI 1.003–1.024, p = 0.013) and combined visceral metastases (HR 13.404, 95% CI 3.706–48.479, p < 0.001; Fig. 2B) were independently associated with worse OS. Regarding treatment toxicity, three patients had vertebral compression fracture (VCF), and no other adverse treatment effects were observed.

## Discussion

The current study evaluated the clinical outcomes of spinal SBRT in patients with sarcomas. The 1-year OS and PFS rates were 80.5% and 31.9%, respectively, and both were significantly associated with disease status at the time of treatment. Patients with solitary spine involvement showed favorable survival outcomes, while those with visceral metastases demonstrated dismal results. For overall patients, the LC rates at 1 and 2 years were 76.4% and 62.9%, respectively, and the irradiated vertebral level was found to be prognostic factors for LC. However, multivariate analyses could not show a correlation between the irradiation dose and LC in this study.

Despite the rarity of sarcoma, a few studies have examined the clinical outcomes of spine SBRT in sarcoma patients (Table 5) [10–13]. In the study by Folkert et al. [10], which included the largest number of lesions, leiomyosarcoma was found to be the most common histology, and favorable clinical outcomes were demonstrated with a median follow-up of 12 months. Although previous studies were not specifically focused on soft tissue sarcoma, leiomyosarcoma remained the predominant histology among spinal sarcoma patients treated with SBRT, with reported 1-year LC and OS rates ranging from 50–88% and 60–70%, respectively. In our study, there was a difference in the patient group as approximately half of the patients had osteosarcoma, known as radioresistant, and only three patients had leiomyosarcoma [16, 17]. Nevertheless, we observed a 1-year LC rate of 76% and an OS rate of approximately 80%, indicating an excellent clinical outcome.

**Table 5:** Results of spine SBRT for sarcoma patients

Study	Time period	Number of patients	Number of lesions	Predominant histology of lesions	Dose fractionation	Median follow-up	Local control	Overall survival
Folkert et al. 10	2005–2012	88	120	Leiomyosarcoma (30%), Other/spindle-cell (18%), Hemangiopericytoma/solitary fibrous tumor (16%)	18–24 Gy/1 fx 24–36 Gy/3–6 fx	12.3 months	1-year 87.9%	1-year 60.6%
Miller et al. 11	2005–2012	18	36	Leiomyosarcoma (32%), Chondrosarcoma (17%), Spindle cell (17%)	Median 16 Gy/1 fx (range, 10–25 Gy/1–5 fx)	15 months	1-year 50%	1-year 60%
Bishop et al. 12	2002–2013	48	66	Leiomyosarcoma (42%), Epithelioid (14%), MFH/UPS (12%)	BED: < 50 Gy_10_ (n = 11), 50–59 Gy_10_ (n = 40), ≥ 60 Gy_10_ (n = 15)	19 months	1-year 81%	1-year 67%
Elibe et al. 13	2001–2013	23	53	Leiomyosarcoma (39%), Ewing’s (13%)	Median 18 Gy/1 fx (range, 10–20 Gy/1 fx)	14 months	Overall 67%	Median 15.5 months
This study	2006–2017	44	75	Osteosarcoma (57%), MPNST (12%)	Median 33 Gy/3 fx (range, 18–45 Gy/1–5 fx)	18.2 months	1-year 76.4%	1-year 80.5%

*MFH* malignant fibrous histiocytomas, *UPS* undifferentiated pleomorphic sarcoma, *MPNST* malignant peripheral nerve sheath tumor

Due to the diverse histologic subtypes of sarcoma, research related to sarcoma has faced challenges [18]. While sarcoma is generally considered to exhibit radioresistance, there may be variability in the radiosensitivity based on histology. In recent years, efforts have been made to calculate radiosensitivity index (RSI) using genomic data [19–22], and Yang and colleagues also applied this approach to soft tissue sarcoma [23], providing RSI values for each histology. Furthermore, Roohani et al. [24] established and explored the radiosensitivity using patient-derived 3D cell cultures, which may reflect the heterogeneity of sarcomas. They reported an apparent difference in radiosensitivity between undifferentiated pleomorphic sarcoma and pleomorphic liposarcoma. Given these findings, we have been curious about whether radioresistance heterogeneity leads to variations in clinical outcomes. Although we reanalyzed the clinical outcomes based on the radiosensitivity of various histologies, following previous reports, we did not observe any significant differences in treatment responses based on their radiosensitivity. Nevertheless, our cohort has limitations; it is both too small and heterogeneous to identify any meaningful differences. We anticipate that future studies will delve further into this inquiry.

Previous studies have generally been unsuccessful in identifying prognostic factors associated with LC. However, in our study, we found that the number of treated vertebral levels was a significant factor influencing LC. Our findings align with previous studies that reported LC rates of 84–88% for single metastatic lesions, as we also demonstrated a high LC rate of 81% for single-level cases [7, 8]. On the other hand, we did not observe a relationship between histology and LC, which is consistent with a previous study that reported the lack of impact of primary tumor histology on treatment outcomes [9].

The dose–response relationship of spinal SBRT for sarcoma patients remains uncertain. Previous studies, as summarized in Table 5, have employed different dose-fractionation regimens. In our study, various doses of BED ranging from 60 to 180 Gy_5_ were administered; however, no statistically significant difference in LC was observed based on the dose. Folkert et al. [10]. conducted a multivariate analysis and found that single fraction SBRT was associated with improved LC. Although they did not directly establish an association between BED and LC, the described median dose implied that the single fraction SBRT had a higher BED of 139.2 Gy_5_ compared to 82.7 Gy_5_ in the hypofractionated SBRT group. Miller et al. [11], while not considering LC as the primary outcome, demonstrated a significant correlation between minimum target dose and unadjusted pain progression.

VCF is one of the significant toxicities following spinal SBRT, with reported rates of up to 36% [25]. However, in our study, VCF was observed in only three patients (6.8%). This discrepancy in rates could be attributed to differences in follow-up periods and the generally poor clinical courses of sarcoma patients compared to those with other primary cancers. Other studies investigating spinal SBRT for sarcoma have reported varying rates of VCF occurrence, ranging from 2.1 to 34.8% [11–13].

We observed 1-year PFS and OS rates of 31.9% and 80.5%, respectively, and identified several factors associated with these survival outcomes. Disease extent at the time of treatment demonstrated a strong association with both PFS and OS. Furthermore, the time since the initial diagnosis and PTV were identified as prognostic variables for PFS and OS, respectively. Despite the generally poor prognosis for patients with spinal sarcoma, we believe that this study offers valuable insights into the management of oligometastasis in the modern era, including the potential for long-term control and identification of prognostic factors for primary and metastatic spinal sarcoma.

In conclusion, spinal SBRT can provide effective LC for primary and metastatic spinal sarcoma. Certain patients with limited disease extent or small target volumes have shown excellent clinical outcomes with long-term intervals through the utilization of spinal SBRT. Although the dose–response relationship remains uncertain, it can be suggested that patients receiving an appropriate SBRT dose may attain a durable response. Therefore, the active consideration of spinal SBRT should be emphasized as it holds the potential to significantly impact the prognosis of patients with oligometastasis.

### Supplementary Information

**Additional file 1: Supplementary Figure 1.** Treatment planning images obtained from a 66-year-old man with angiosarcoma metastases.

## Data Availability

The datasets used in the current study are available from the corresponding author on reasonable request.

## Abbreviations

SBRT: Stereotactic body radiation therapy
GTV: Gross tumor volume
MRI: Magnetic resonance imaging
PTV: Planning target volume
LC: Local control
CT: Computed tomography
BED: Biological equivalent dose
PFS: Progression-free survival
OS: Overall survival
HR: Hazard ratio
CI: Confidence interval
RSI: Radiosensitivity index
VCF: Vertebral compression fracture
