# Correction: Treatment outcomes of stereotactic body radiation therapy for primary and metastatic sarcoma of the spine

**DOI:** 10.1186/s13014-023-02358-6

**Published:** 2023-11-07

**Authors:** Eunji Kim, Mi-Sook Kim, Eun Kyung Paik, Ung-Kyu Chang, Chang-Bae Kong

**Affiliations:** 1 Department of Radiation Oncology, Seoul Metropolitan Government - Seoul National University Boramae Medical Center, Seoul, Republic of Korea; 2 Department of Radiation Oncology, Korea Institute of Radiological and Medical Sciences, Seoul, Republic of Korea; 3 Department of Neurosurgery, Korea Institute of Radiological and Medical Sciences, Seoul, Republic of Korea; 4 Department of Orthopedic Surgery, Korea Institute of Radiological and Medical Sciences, 75, Nowon-ro, Nowon-gu, Seoul, 01812 Republic of Korea

**Radiation Oncology (2023) 18:156**

10.1186/s13014-023-02346-w

After publication of this article [1], the authors reported that in this article the funding from ‘National Research Foundation of Korea (NRF) grant funded by the Ministry of Science & ICT’ was omitted.

The original article [1] has been corrected.
