# Supplementary material for: Treatment outcomes of stereotactic body radiation therapy for primary and metastatic sarcoma of the spine
Source: Radiat Oncol. 2023 Sep 22;18:156. doi: 10.1186/s13014-023-02346-w (PMC10514933; doi:10.1186/s13014-023-02346-w)
Supplement: Supplementary file 1 — Additional file 1: Supplementary Figure 1. Treatment planning images obtained from a 66-year-old man with angiosarcoma metastases. [file 13014_2023_2346_MOESM1_ESM.pptx]

## Slide 1
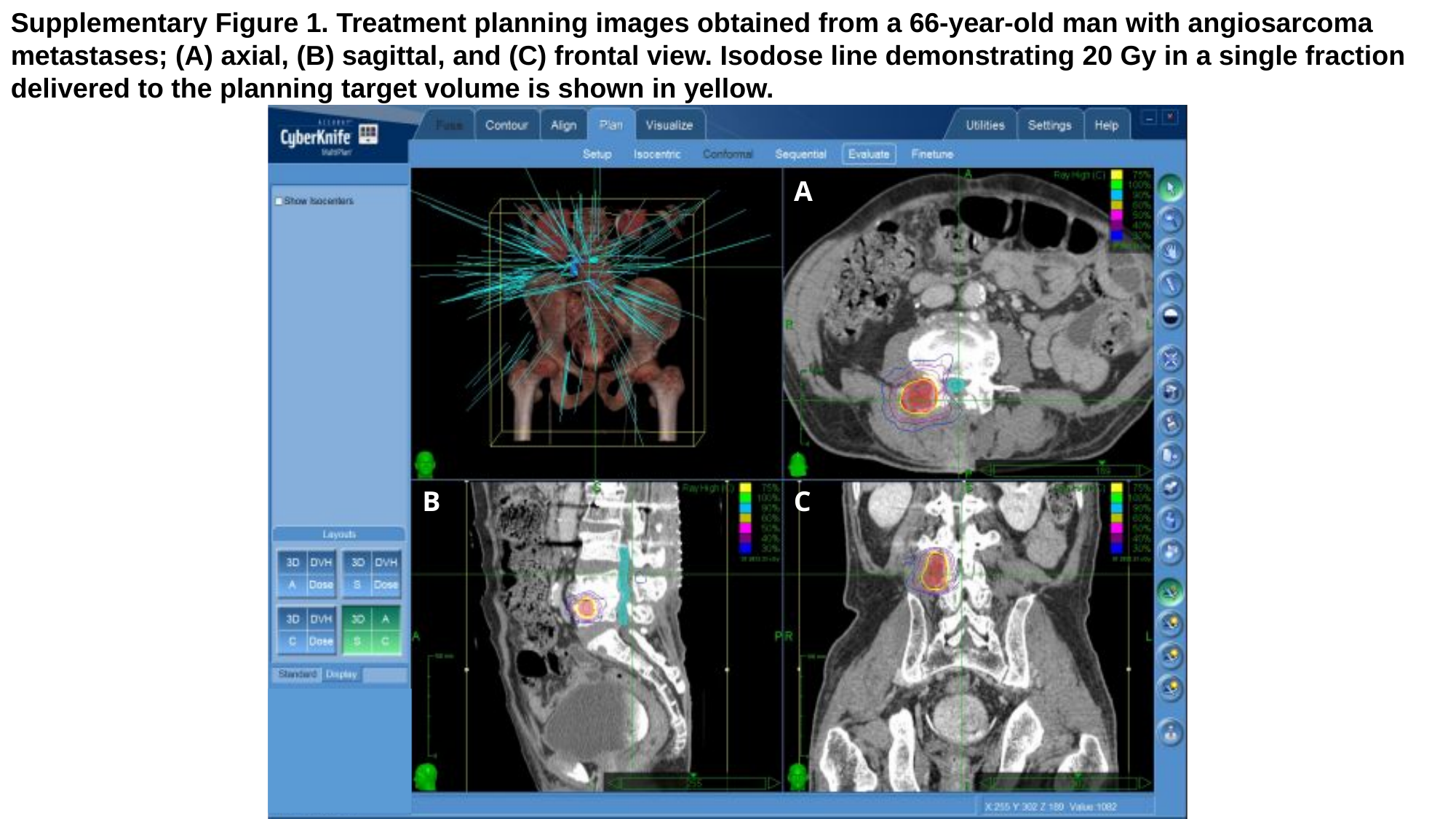

Supplementary Figure 1. Treatment planning images obtained from a 66-year-old man with angiosarcoma metastases; (A) axial, (B) sagittal, and (C) frontal view. Isodose line demonstrating 20 Gy in a single fraction delivered to the planning target volume is shown in yellow.
A
B
C
